# An Isocaloric High-Fat Diet Regulates Partially Genetically Determined Fatty Acid and Carbohydrate Uptake and Metabolism in Subcutaneous Adipose Tissue of Lean Adult Twins

**DOI:** 10.3390/nu15102338

**Published:** 2023-05-16

**Authors:** Michael Kruse, Silke Hornemann, Anne-Cathrin Ost, Turid Frahnow, Daniela Hoffmann, Andreas Busjahn, Martin A. Osterhoff, Bettina Schuppelius, Andreas F. H. Pfeiffer

**Affiliations:** 1 Department of Endocrinology, Diabetes and Nutrition, Charité—Universitätsmedizin Berlin, Corporate Member of Freie Universität Berlin, Humboldt-Universität zu Berlin, and Berlin Institute of Health, Charitéplatz 1, 10117 Berlin, Germany; 2 German Institute of Human Nutrition Potsdam-Rehbruecke, Arthur-Scheunert-Allee 114-116, 14558 Nuthetal, Germany; 3 Health TwiSt GmbH, Robert-Rössle-Strasse 10, 13125 Berlin, Germany; 4 German Center for Diabetes Research (DZD), Ingolstädter Landstrasse 1, 85764 Neuherberg, Germany

**Keywords:** isocaloric high fat diet, adipose tissue, nutrient transport, heritability, twins

## Abstract

Background: The dysfunction of energy metabolism in white adipose tissue (WAT) induces adiposity. Obesogenic diets that are high in saturated fat disturb nutrient metabolism in adipocytes. This study investigated the effect of an isocaloric high-fat diet without the confounding effects of weight gain on the gene expression of fatty acid and carbohydrate transport and metabolism and its genetic inheritance in subcutaneous (s.c.) WAT of healthy human twins. Methods: Forty-six healthy pairs of twins (34 monozygotic, 12 dizygotic) received an isocaloric carbohydrate-rich diet (55% carbohydrates, 30% fat, 15% protein; LF) for 6 weeks followed by an isocaloric diet rich in saturated fat (40% carbohydrates, 45% fat, 15% protein; HF) for another 6 weeks. Results: Gene expression analysis of s.c. WAT revealed that fatty acid transport was reduced after one week of the HF diet, which persisted throughout the study and was not inherited, whereas intracellular metabolism was decreased after six weeks and inherited. An increased inherited gene expression of fructose transport was observed after one and six weeks, potentially leading to increased de novo lipogenesis. Conclusion: An isocaloric dietary increase of fat induced a tightly orchestrated, partially inherited network of genes responsible for fatty acid and carbohydrate transport and metabolism in human s.c. WAT.

## 1. Introduction

The dysfunction of energy metabolism in white adipose tissue (WAT) is responsible for the development of obesity and subsequently chronic diseases, such as metabolic syndrome, diabetes mellitus type 2 and cardiovascular diseases [1]. Alarming epidemiological data indicate that by the year 2025, obesity will affect 21% of women and 18% of men worldwide [2]. Major risk factors promoting obesity are overeating of unhealthy hypercaloric diets, a sedentary lifestyle and genetic predisposition [3]. Adipose tissue generates and stores energy mainly from dietary fats and carbohydrates depending upon availability [4]. Under healthy, non-pathological conditions, the uptake and metabolism of fats and carbohydrates in adipocytes are tightly regulated and assure balanced lipid storage [3]. During obesity, these mechanisms are disturbed, which can result in systemic insulin resistance and inflammation [3]. The majority of studies that investigated nutrient uptake and energy metabolism in adipocytes are performed once overt obesity has already occurred and/or after consuming different types of hypercaloric diets. However, periods of consuming unhealthy diets without weight change might precede the development of obesity. In light of today’s eating habits, studies that investigate nutrient uptake mechanisms and their potential genetic determination in adipose tissue prior to the manifestation of obesity are needed and relevant for understanding the development of pathological energy metabolism and for developing concepts of personalized nutrition.

In adipocytes, free fatty acids (FFAs) are hydrolyzed from triglycerides of plasma lipoproteins by lipoprotein lipase (LPL) [5,6] and transported into the cell via the transmembrane receptor fatty acid translocase (FAT)/cluster of differentiation 36 (CD36) [7]. Once FFAs are high in the bloodstream, CD36 expression increases in adipocytes [8]. CD36 is believed to be involved in the development of adipose tissue insulin resistance in humans and is increased in adipocytes during obesity [9]. In the adipocyte, FFAs are re-esterified and stored as triglycerides in lipid droplets.

Carbohydrates mainly enter the adipocyte by specific transmembrane glucose transporter (GLUT) proteins that allow for the transport of monosaccharides across the plasma membrane via facilitated diffusion [10]. GLUT4 is the major glucose transporter in adipose tissue and is stored in intracellular vesicles that translocate to the plasma membrane upon insulin stimulation, allowing for a very rapid uptake of glucose into the cell [11]. GLUT1 is a ubiquitous glucose transporter that maintains basal glucose uptake [12]. In the cell, glucose undergoes glycolysis and its metabolite pyruvate enters the mitochondria and is further oxidized to acetyl coenzyme A, which, in turn, is utilized by fatty acid synthase (FASN) for de novo fatty acid synthesis [13]. FASN in adipose tissue is increased during obesity [14] and by consuming diets containing high amounts of carbohydrates [15]. It was shown that adipocytes also utilize fructose that enters the cell through GLUT5, which exclusively transports fructose [16]. In vitro studies showed that the lipogenesis of adipocytes was enhanced in the presence of increased fructose concentration [17]. Fructose is metabolized either to pyruvate and subsequently used for de novo fatty acid synthesis or to glycerol-3-phosphate, which is required for fatty acid re-esterification. However, whether GLUT5-mediated fructose metabolism in human adipose tissue is affected by other diets, e.g., a diet high in saturated fats without an excess of fructose, is currently unknown.

Heritability studies in twins suggested that both genetic and environmental factors contribute to the development of metabolic syndrome [18]. In this study, we investigated the early effects of a switch from an isocaloric healthy high-carbohydrate, low-saturated-fat diet to an isocaloric low-carbohydrate, high-saturated-fat diet on the gene expression of fatty acid and carbohydrate uptake and metabolism in subcutaneous adipose tissue in a cohort of 34 monozygotic and 12 dizygotic pairs of healthy twins. We aimed to evaluate the heritability of the gene expression in adipocytes without the confounding effects of weight gain and to explore early genetic markers that might precede the development of obesity in this human cohort.

## 2. Experimental Section

### 2.1. Study Participants

Pairs of twins were recruited either from a twin register (HealthTwiSt, Berlin, Germany) or by advertisements in the local newspapers. A total of 34 monozygotic and 12 dizygotic pairs of twins (a total of 92 individuals), aged 18–70 years, a body mass index (BMI) of 18.5–29 kg/m^2^, with a difference in BMI not higher than 3 kg/m^2^ were included in the study. The study design was explained in detail and all participants gave their written informed consent before they participated in the study. The study protocol was approved by the local ethical committee of the Charité University of Medicine, Berlin, Germany (protocol code EA4/021/09, date of approval 26 September 2009) and was in accordance with the Declaration of Helsinki of 1975, as revised in 2013. This study was registered at ClinicalTrials.gov (NCT01631123).

At the initial visit, a physical examination was performed and fasted blood (12 h fast overnight) was collected for analysis of a red blood count, serum lipids, glucose and insulin. A 75 g oral glucose tolerance test was performed to exclude impaired glucose homeostasis or diabetes mellitus. Indirect calorimetry was used to measure the resting energy expenditure (REE) and the physical activity level (PAL) was assessed using a questionnaire. Body weight (kg), height (m), and waist and hip circumferences (cm) were taken and the body mass index (BMI) and waist-to-hip-ratio were calculated. Participants were asked to fill out a dietary food record for five days (four weekdays, one weekend day) prior to the study to evaluate their dietary habits. Exclusion criteria of this study were as followed: a medical history of consuming chronic illnesses, diabetes mellitus, change in body weight >3 kg within three months prior to the study, drugs influencing metabolic homeostasis, lipid metabolism, liver metabolism or inflammation (e.g., systemic corticosteroids), a difference in BMI higher than 3 kg/m^2^ between twins, or an unwillingness to follow the protocol or to give informed consent. The baseline characteristics of monozygotic and dizygotic pairs of twins are shown in Table 1.

### 2.2. Study Design

**Figure 1:**
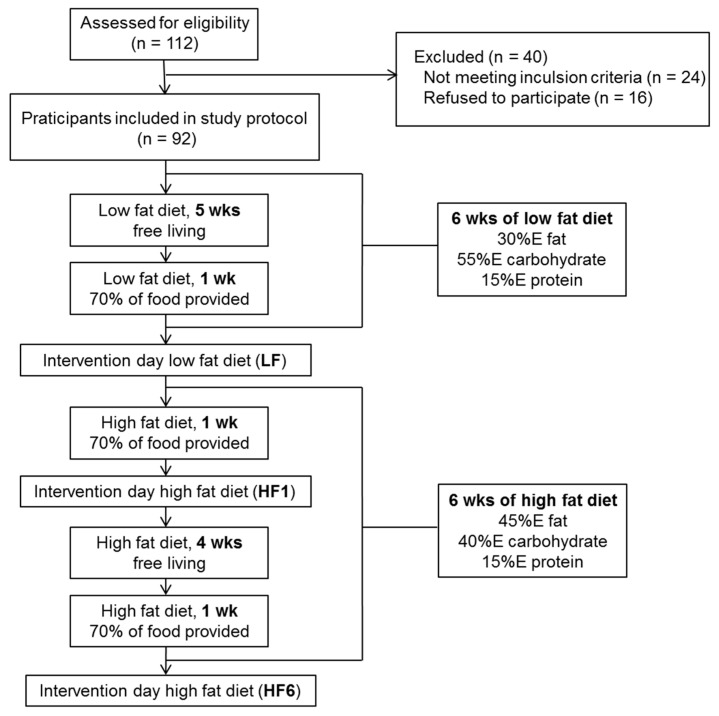
Timeline of the study. After the assessment for eligibility, the participants received an isocaloric high-carbohydrate, low-fat diet for six weeks, followed immediately by an isocaloric low-carbohydrate, high-fat diet for another six weeks. Anthropometric measurements, blood tests and a biopsy of abdominal subcutaneous adipose tissue for the gene expression analysis were performed after six weeks on the isocaloric high-carbohydrate, low-fat diet (LF) and after one week (HF1) and six weeks (HF6) on the isocaloric low-carbohydrate, high-fat diet.

The timeline of the study is shown in Figure 1. Prior to the dietary intervention, individual energy requirements were calculated based on the participants’ REE and PAL, and individual goals for consuming carbohydrates, fat and protein were determined to achieve the consumption of an isocaloric diet. All participants started with six weeks of a high-carbohydrate, low-fat diet containing 55%E carbohydrates, 30%E fat and 15%E protein. During the first five weeks, participants were instructed to consume the diet in a free-living mode. They received a list with detailed information on 94 food items, how to exchange and combine them and which foods to prefer or avoid. To ensure compliance, participants were given intensive counseling and support by the nutritionist and were asked to complete a five-day food record after three weeks on the diet. For the last week on the high-carbohydrate, low-fat diet, all participants were provided with approximately 70 percent of the food and detailed meal plans to ensure a standardized dietary pattern. Again, participants received intensive counseling from the nutritionist and were asked to complete a six-day food record. Immediately after the six weeks of the high-carbohydrate, low-fat diet, participants underwent the first day of investigation (LF). Fasted blood was drawn for routine laboratory markers. Anthropometric measurements (body weight, BMI, waist-to-hip ratio) were performed and the total body fat content was determined. A biopsy of abdominal subcutaneous adipose tissue was performed for the gene expression analysis. From then on, the diet was changed to a low-carbohydrate, high-fat diet containing 40%E carbohydrates, 45%E fat and 15%E protein for an additional six weeks. Again, 70 percent of food was provided for the first week of this period and participants were asked to complete a six-day food record. After this week, the participants underwent the second intervention day to assess the short-term effects of the low-carbohydrate, high-fat diet (HF1). On the HF1 intervention day, the participants received intensive nutritional counseling. For the next four weeks, the participants were instructed to consume the diet in a free-living mode. After a total of three weeks on the low-carbohydrate, high-fat diet, they were asked to complete a five-day food record and had a detailed telephone counseling session with the nutritionist. For the last week of the low-carbohydrate, high-fat diet, the participants were again handed out approximately 70 percent of the appropriate food, received intensive nutritional counseling in person in our unit and detailed meal plans, and were asked to complete a six-day food record. At the end of the entire low-carbohydrate, high-fat period, a third intervention day was performed (HF6). On each intervention day, all tests were performed at the same time of the day to ensure comparability.

### 2.3. Blood Analyses

Routine serum parameters (red blood count, total cholesterol, HDL, triglycerides, free fatty acids) were measured using standard techniques in a certified laboratory for clinical chemistry at the German Institute of Human Nutrition after an overnight fast at 8:00 am. LDL cholesterol was calculated from the above data. Serum insulin was measured using commercially available ELISA (Mercodia, Uppsala, Sweden). Glucose concentrations were measured in venous serum samples (ABX Pentra 400; ABX Diagnostics, Montpellier, France).

### 2.4. Adipose Tissue Biopsy

On each investigation day, a biopsy of the subcutaneous adipose tissue fat pad was performed laterally to the umbilicus via needle aspiration. After local anesthesia (1% lidocaine), a needle connected to a syringe was inserted into the adipose tissue. A vacuum was applied and approximately 1 g of tissue was taken. The tissue was rinsed briefly with 0.9% NaCl saline, immediately snap-frozen in liquid nitrogen and stored at −80° C for further analyses.

### 2.5. Gene Expression Analyses

mRNA expression analysis was performed in adipose tissue using quantitative real-time PCR as described previously [19]. Approximately 500 mg of tissue was homogenized using a speed mill (Speed Mill 12, Analytik Jena, Jena, Germany) in QIAzol^®^ lysis buffer provided with the RNeasy^®^ Lipid Tissue Midi Kit (Qiagen, Hilden, Germany). mRNA was extracted by following the manufacturer’s instructions for the kit. The purity of the mRNA was tested using a spectrophotometer (NanoDrop Technologies, Wilmington, NC, USA). mRNA was used only if the E_280_/E_260_ quotient was between 1.8 and 2.0 and the E_260_/E_230_ quotient was between 1.8 and 2.2. To assess the quality of the mRNA, the RNA integrity number (RIN) was determined using a 2100 Bioanalyzer (Agilent Technologies, Santa Clara, CA, USA). If an RIN > 8.0 was obtained, the mRNA was accepted for further processing. cDNA was synthesized using a High-Capacity cDNA Reverse Transcription Kit (Applied Biosystems Inc., Forster City, CA, USA). Gene expression analysis was performed via quantitative real-time PCR using Power-SYBR^®^Green-PCR_Master Mix (Applied Biosystems, Thermo Fisher Scientific Inc., Waltham, MA, USA) and the Applied Biosystems^®^ ViiA7^TM^ real-time PCR System (Waltham, MA, USA). Analyses were performed in triplicates. To verify the purity of the PCR products and exclude non-specific PCR products, e.g., primer dimers, a melting curve analysis was performed and PCR products were separated on a 2% agarose gel. Gene expression analysis was performed for adiponectin (*adipoq*), carnitine palmitoyltransferase 1A (*cpt1a*), fatty acid translocase (FAT)/cluster of differentiation 36 (*cd36*), fatty acid synthase (*fasn*), GLUT1 (*slc2a1*), GLUT4 (*slc2a4*), GLUT5 (*slc2a5*), GLUT8 (*slc2a8*), peroxisome proliferator-activated receptor gamma (*pparg*), peroxisome proliferator-activated receptor gamma, coactivator 1 alpha (*ppargc1a*), pyruvate dehydrogenase kinase, isozyme 4 (*pdk4*), interleukin-6 (*il6*), lipoprotein lipase (*lpl*) and tumor necrosis factor alpha (*tnfa*). The constitutively expressed gene ribosomal protein L32 (*rpl32*) was used as a loading control. A list of primer sequences is shown in Table S1. Gene sequences for primer design were determined using the National Institute of Health (NIH), USA, website www.ncbi.nlm.nih.gov/sites/entrez, and primers were designed using Primer Express 2.0 (Applied Biosystems, Carlsberg, USA). Primer pairs were selected if they had similar melting points, were covering intron-spanning regions and had no ability to form dimers. The specificity of primers was tested using the Basic Local Alignment Search Tool (BLAST) (http://blast.ncbi.nlm.nih.gov/Blast.cgi). Gene expression was determined using a standard curve. Target genes were normalized to the constitutively expressed gene *rpl32*.

### 2.6. Statistical Analysis and Quantitative Genetics

Before the analysis, data were tested for plausibility. Values outside of the threefold interquartile range were declared as extreme outliers and not considered for further analysis.

The Kolmogorov–Smirnov test was used to assess variables for normal distribution. Continuous variables with skewed distribution were natural logarithm (ln)-transformed. One-way or repeated-measures ANOVA followed by a Bonferroni post hoc test was used to compare mean values for continuous data. To verify significant results for non-normally distributed data, the Kruskal–Wallis test was used. All data are given as the mean ± standard deviation (SD). Statistical analysis was performed using SPSS 20.0 (SPSS, Chicago, IL, USA) and the integrated development environment RStudio (version 0.97.336), which is based on R (version 3.0.0). A two-sided *p*-value < 0.05 was considered significant. The correlation analysis was performed with Spearman’s rank correlation coefficient (Spearman’s rho).

For the estimation of heritability, the “ACE” structural equation model was applied. This covariance analysis relied on comparing the degree of concordance within and between monozygotic versus dizygotic twin pairs and decomposed the proportion of variance into (A) additive genetic influences and (C) common environmental and (E) individual environmental influences. The “ACE” model was calculated using R 2.15.0 plus the OpenMX package. Genotype frequencies were analyzed for deviation from the Hardy–Weinberg equilibrium with a chi-squared test using R 3.1.2 plus the Hardy–Weinberg package 1.5.5.

## 3. Results

### 3.1. Body Weight, BMI, Serum Lipids, Glucose and Insulin

Over the twelve weeks of the dietary intervention, body weight and BMI did not change for monozygotic twins (MZ) or dizygotic twins (DZ) (Table 2). Serum total cholesterol increased after six weeks on the low-carbohydrate, high-fat diet (HF6) compared with six weeks on the high-carbohydrate, low-fat diet (LF); however, this reached significance in MZ (1.09-fold, *p* = 0.021) only (Table 2). Serum-free fatty acids decreased significantly at HF6 compared with LF 18.5% in MZ (*p* = 0.004) and 15.5% in DZ (*p* = 0.008) (Table 2). Blood glucose and insulin levels did not change significantly over the time of the intervention. When calculating the HOMA-IR, we saw a significant increase at HF1 and HF6 for MZ and at HF1 for DZ compared with LF. However, despite the significance, the numerical increase in HOMA-IR was low. We did not observe any changes in serum LDL cholesterol, HDL cholesterol or triglycerides (Table 2).

### 3.2. Gene Expression in Human s.c. Adipose tissue

**Figure 2:**
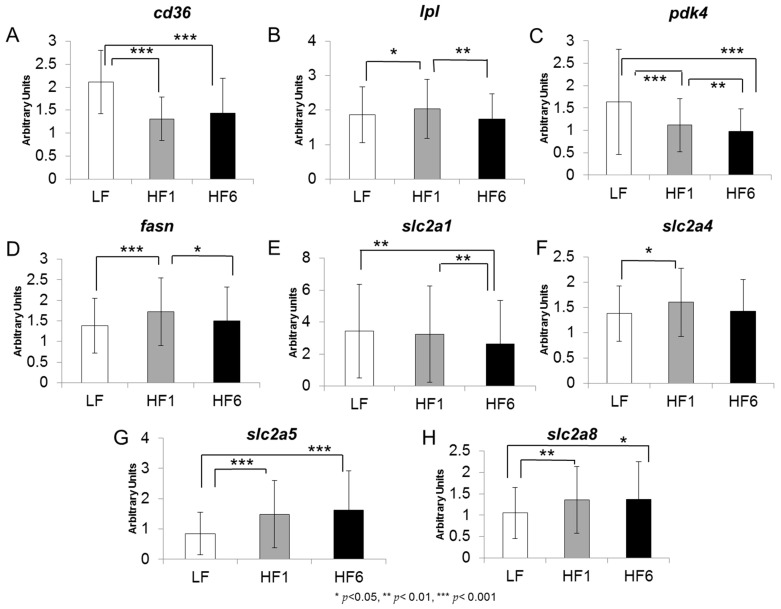
Gene expression levels of *cd36* (**A**), *lpl* (**B**), *pdk4* (**C**), *fasn* (**D**), *slc2a1* (**E**), *slc2a4* (**F**), *slc2a5* (**G**) and *slc2a8* (**H**) in s.c. adipose tissue. The gene expression was analyzed in a biopsy of adipose tissue after six weeks on the isocaloric high-carbohydrate, low-fat diet (LF) and after one week (HF1) and six weeks (HF6) on the isocaloric low-carbohydrate, high-fat diet. Values are given as mean ± SD; * *p* < 0.05, ** *p* < 0.01, *** *p* < 0.001; *n* = 80–84.

**Figure 3:**
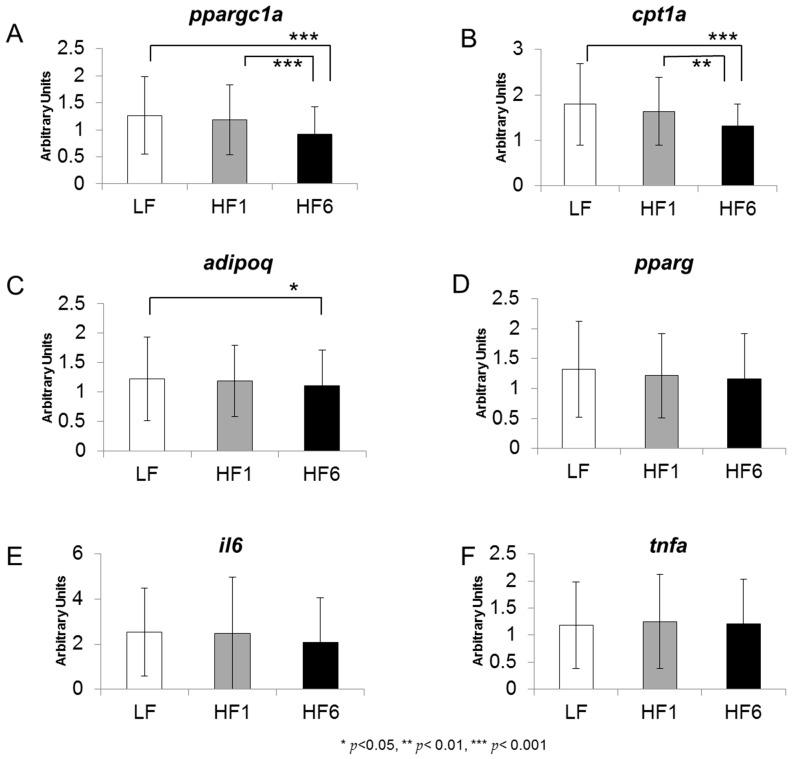
Gene expression levels of *ppargc1a* (**A**), *cpt1a* (**B**), *adipoq* (**C**), *pparg* (**D**), *il6* (**E**) and *tnfa* (**F**) in s.c. adipose tissue. The gene expression was analyzed in a biopsy of adipose tissue after six weeks on the isocaloric high-carbohydrate, low-fat diet (LF) and after one week (HF1) and six weeks (HF6) on the isocaloric low-carbohydrate, high-fat diet. Values are given as mean ± SD; * *p* < 0.05, ** *p* < 0.01, *** *p* < 0.001; *n* = 80–84.

The overall gene expressions for all individuals in the study are shown in Figure 2 and Figure 3. The gene expression of *cd36* was strongly decreased by 37.8% after one week (HF1) and by 31.8% after six weeks (HF6) of the isocaloric high-fat diet compared with LF (for both *p* < 0.001) (Figure 2A). For the *lpl* gene expression, a 1.10-fold increase was seen at HF1 compared with LF (*p* = 0.025) and was significantly decreased at HF6 (*p* = 0.002 compared with HF1) (Figure 2B).

A significant decrease in the gene expression of *pdk4* of 31.7% was observed at HF1 (*p* < 0.001 compared with LF) and was even more downregulated by 40.1% at HF6 (*p* < 0.001 compared with LF and *p* = 0.002 compared with HF1) (Figure 2C). An increase in the gene expression was observed at HF1 for *fasn* (1.24-fold, *p* < 0.001), and significantly decreased by 12.5% at HF6 (*p* = 0.019 compared with HF1) (Figure 2D).

The gene expression of *slc2a1* was not affected by the acute switch from the isocaloric high-carbohydrate, low-fat diet to the isocaloric low-carbohydrate, high-fat diet, but decreased by 23.5% after six weeks (*p* = 0.001 compared with LF and HF1) (Figure 2E). The *slc2a4* gene expression showed a 1.16-fold increase at HF1 compared with LF (*p* = 0.042, Figure 2F). However, this increase disappeared at HF6. For *slc2a5*, we observed a strong upregulation of the gene expression at HF1 compared with LF (1.77-fold, *p* = 0.001) that further increased at HF6 (1.92-fold compared with LF, *p* = 0.001) (Figure 2G). The *slc2a8* gene expression showed a marked increase at HF1 compared with LF (1.29-fold, *p* = 0.001) that persisted at HF6 (1.31-fold increase compared with LF, *p* = 0.038) (Figure 2H).

For *ppargc1a* and *cpt1a*, we did not observe any changes in the gene expression after one week of the diet rich in saturated fat, but a significant decrease after six weeks of 12.0% for *ppargc1a* (*p* < 0.001 compared with LF and HF1) and of 26.8% for *cpt1a* (*p* < 0.001 compared with LF and *p* = 0.002 compared with HF1) (Figure 3A,B).

For the gene expression of *adipoq*, a slight decrease of 8.9% was observed after six weeks of the diet rich in saturated fat (*p* = 0.018 compared with LF, Figure 3C).

We did not observe any significant differences in the gene expressions for *pparg*, *il6* and *tnfa* (Figure 3D–F).

Table 3 shows a synopsis of the genes investigated in this study, their functions and their regulations after exposure to the low-carbohydrate, high-fat diet.

### 3.3. Determination of the Heritability of Gene Expression in s.c. Adipose Tissue

**Figure 4:**
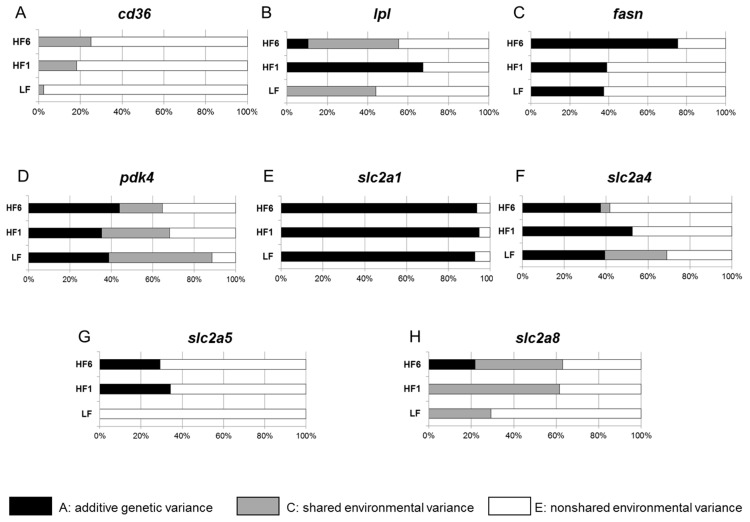
Heritability of the gene expressions of *cd36* (**A**), *lpl* (**B**), *pdk4* (**C**), *fasn* (**D**) *slc2a1* (**E**), *slc2a4* (**F**), *slc2a5* (**G**) and *slc2a8* (**H**) in s.c. adipose tissue. The additive genetic influences and the common environmental and individual environmental influences of expression for the indicated genes were estimated.

For the gene expression of *cd36*, no heritability was observed for all time points (Figure 4A). For *lpl* gene expression, no heritability was seen at LF; however, a strong heritability was seen after one week on the high-fat diet (A = 0.673 at HF1), while heritability was weak at HF6 (A = 0.105) (Figure 4B).

For *fasn* gene expression, a moderate heritability was observed prior to the HF diet at LF (A = 0.375) and did not change at HF1 (A = 0.390); however, this strongly increased to A = 0.754 at HF6 (Figure 4C). The *pdk4* gene expression showed a moderate heritability at LF (A = 0.388) that did not significantly change over the time of intervention (A = 0.354 at HF1 and A = 0.440 at HF6) (Figure 4D).

For the *slc2a1* gene expression, a strong heritability with only small changes was observed throughout the study: A = 0.927 at LF, A = 0.947 at HF1 and A = 0.936 at HF6 (Figure 4E). The heritability of the *slc2a4* gene expression was moderate at LF (A = 0.394) and transiently increased to A = 0.525 at HF1, followed by a decrease at HF6 (A = 0.373) (Figure 4F). We did not see any heritability of the *slc2a5* gene expression at LF (Figure 4G). However, the switch from the high-carbohydrate, low-fat diet to the low-carbohydrate, high-fat diet induced an increase in heritability of *slc2a5* at HF1 (A = 0.340) and persisted to HF6 (A = 0.290). For *slc2a8*, no heritability of the gene expression was observed at LF or HF1; however, moderate heritability was seen at HF6 (A = 0.220) (Figure 4H).

**Figure 5:**
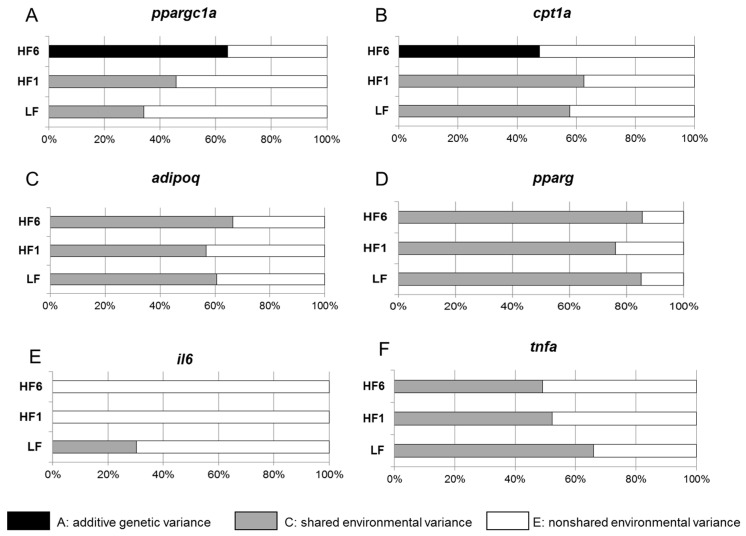
Heritability of the gene expression of *ppargc1a* (**A**), *cpt1a* (**B**), *adipoq* (**C**), *pparg* (**D**), *il6* (**E**) and *tnfa* (**F**) in s.c. adipose tissue. The additive genetic influences and the common environmental and individual environmental influences of expression for the indicated genes were estimated.

Remarkably, expression of the mitochondrial genes *ppargc1a* and *cpt1a* did not show any heritability at LF or HF1, but a robust heritability was found at HF6 (A = 0.643 for *ppargc1a* and A = 0.475 for *cpt1a*) (Figure 5A,B).

For *adipoq*, *pparg*, *il6* and *tnfa*, no heritability of the gene expression was observed during the study (Figure 5C–F).

### 3.4. Correlation Analysis of the Gene Expression in s.c. Adipose Tissue

**Figure 6:**
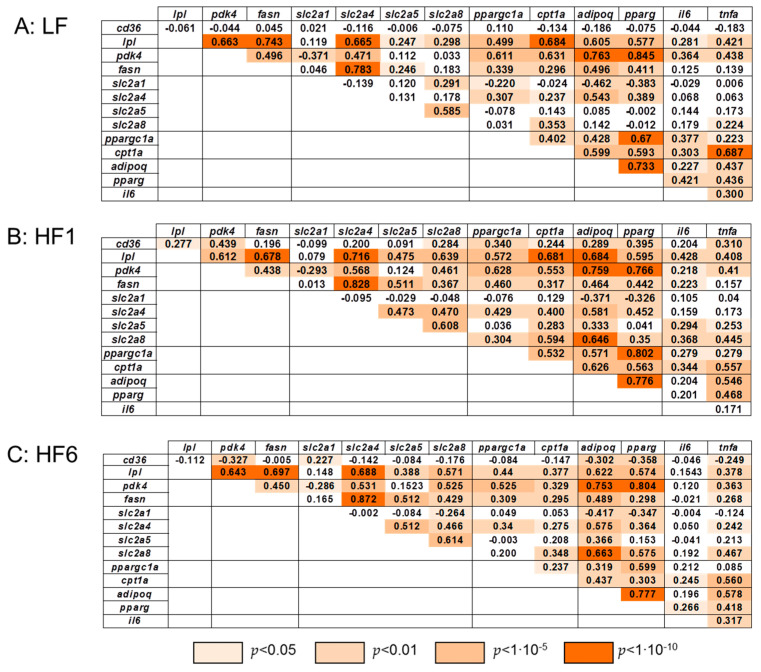
Correlation of gene expression in s.c. adipose tissue. Spearman’s rank correlation coefficients for gene expressions in adipose tissue after six weeks on the isocaloric high-carbohydrate, low-fat diet (LF, (**A**)) and after one week (HF1, (**B**)) and six weeks (HF6, (**C**)) on the isocaloric low-carbohydrate, high-fat diet.

Figure 6 shows Spearman’s correlation coefficients for the gene expression over the time of dietary intervention at LF, HF1 and HF6.

At LF, we observed no correlations for the *cd36* gene expression with any other gene expression. However, exposure to the isocaloric low-carbohydrate, high-fat diet resulted in moderate positive correlations of the gene expression of *cd36* with *pdk4*, *ppargc1a*, *adipoq, pparg* and *tnfa* and mild positive correlations of the gene expressions for *lpl*, *slc2a8* and *cpt1a*. Strikingly, all these positive correlations disappeared at HF6 and turned into negative correlations of the gene expression for *cd36* with *pdk4*, *adipoq*, *pparg* and *tnfa*. Only a mild positive correlation was observed for *cd36* with *slc2a1* gene expression.

The *lpl* gene expression exhibited strong positive correlations with the gene expressions of *pdk4*, *fasn*, *slc2a4*, *ppargc1a*, *cpt1a*, *adipoq* and *pparg* at LF, which did not change much during the time of intervention, whereas a mild and moderate positive correlation was seen for the gene expression of *lpl* with *slc2a5* and *slc2a8* at LF that strongly increased at HF1 and HF6.

For *slc2a1*, no significant positive correlation of the gene expression was observed with any other genes investigated, except moderately with *slc2a8* at HF1 and mildly with *cd36* at HF6.

The *slc2a5* gene expression was mildly positively correlated with the gene expression of *lpl* and *fasn* at LF, which strongly increased once the isocaloric low-carbohydrate, high-fat diet was applied for one (HF1) and six (HF6) weeks. Interestingly, the *slc2a5* and *slc2a8* gene expressions showed a strong positive correlation at LF, which did not change throughout the time of intervention.

The *slc2a8* gene expression was moderately positively correlated with the *cpt1a* gene expression at LF, which increased to a strong correlation at HF1 and decreased back to a moderate correlation at HF6. For the *slca8* gene expression, a mild positive correlation with *tnfa* was seen at LF, whereas moderate positive correlations were seen for *il6* and *tnfa* at HF1, which persisted until HF6 for the *tnfa* gene expression only.

For the mitochondrial genes *cpt1a* and *ppargc1a*, a strong positive correlation of the gene expression was observed at LF and HF1, which decreased to a moderate positive correlation at HF6. As expected, we always saw a very strong positive correlation of the gene expressions for *adipoq* and *pparg* throughout the study.

A moderate positive correlation was observed for the *il6* and *tnfa* gene expressions at LF and HF6 that interestingly transiently disappeared at HF1.

## 4. Discussion

White adipose tissue (WAT) adapts its molecular processes for storing or releasing energy depending on the available nutrients. The novelty of this study was that a sudden switch from an isocaloric high-carbohydrate, low-fat diet (LF) to an isocaloric low-carbohydrate, high-fat diet (HF) orchestrated the expression of the key genes involved in the transport and metabolism of fatty acids and carbohydrates in human s.c. WAT. Moreover, our study revealed that the expressions of certain genes in response to this nutritional challenge were genetically determined.

We observed a robust decrease in the *cd36* gene expression after one week of the switch from an isocaloric high-carbohydrate, low-fat diet to an isocaloric low-carbohydrate, high-fat diet that persisted until week six on that diet. CD36 is important for fatty acid uptake and release in adipocytes [8] and its expression is increased in adipose tissue in human obesity [9]. Studies in rodents showed that 50% of fatty acid uptake in adipose and muscle tissue is mediated by CD36 [20]. However, a clinical study demonstrated that fatty acid uptake is diminished in adipose tissue in humans with CD36 deficiency [21]. The decrease in the *cd36* gene expression in our study was unexpected because of the sudden increase in dietary saturated fat. However, it seems that not only the increase in fatty acids but also the demand for energy was important for the adipose *cd36* gene expression. Since individuals in our study received an isocaloric diet, it is very likely that no extra energy was stored in adipose tissue since we did not observe any increase in body weight (Table 2). A study where 50 obese individuals received either a hypocaloric low-fat or a hypocaloric high-fat diet for 10 weeks showed that the *cd36* gene expression was significantly decreased in both groups at the end of the study [22].

For the gene expression of lipoprotein lipase (*lpl*), which hydrolyses triglycerides from chylomicrons, VLDL and triglyceride-rich lipoproteins [5], we saw a slightly significant increase in the *lpl* gene expression in s.c. WAT after one week of the high-fat diet, but this significantly decreased in the long term after six weeks on the high-fat diet. *Lpl* is regulated in s.c. WAT by dietary macronutrients [5]. In humans, it was shown that a diet rich in carbohydrates increases the *lpl* gene expression of sc. WAT much more strongly than a diet high in fat [23].

No heritability of the gene expression was seen at LF for *cd36* and *lpl*. For *cd36*, this persisted throughout our study. Remarkably, the transient increase in the *lpl* gene expression was highly heritable since we observed a strong inheritance of 67% at HF1. A previous study that investigated lipoprotein lipase activity after exercise in twins suggested the genetic determination of lipoprotein lipase activity in adipose tissue [24]. Thus, despite the importance of both *lpl* and *cd36* for fatty acid uptake in WAT, the regulation of the expression of these genes and their heritability were regulated differently.

The release of fatty acids into the blood during lipolysis is also mediated by CD36 [8]. However, fatty acids are not completely released into circulation and are partially re-esterified into triglycerides [25]. Re-esterification requires the synthesis of glycerol-3-phosphate. Glycerol-3-phosphate is generated in a pathway that initially metabolizes pyruvate to oxaloacetate. Oxaloacetate is converted to phosphoenolpyruvate, which is a precursor of glycerol-3-phosphate, by phosphoenolpyruvate carboxykinase (PEPCK) [26]. Pyruvate dehydrogenase kinase 4 (PDK4) inhibits pyruvate dehydrogenase complexes, leading to increased synthesis of oxaloacetate from pyruvate [27]. Similar to *cd36*, we observed a robust decrease in the *pdk4* gene expression in s.c. WAT at HF1 and HF6, indicating decreased re-esterification in s.c. WAT. However in contrast to *cd36*, we observed heritability for the *pdk4* gene expression which did not change at all three time points. Interestingly, the *cd36* and *pdk4* gene expressions were not correlated at LF but were significantly positively correlated at HF1, which turned into a significant negative correlation at HF6. This indicates that the stressor of the isocaloric low-carbohydrate, high-fat diet acutely coordinated the gene expressions of *cd36* and *pdk4*, but in the long-term adaptation phase, other factors (e.g., heritability) might be responsible for gene expression.

Coordination of the gene expression, which occurred only transiently, was strikingly observed between *cd36* and the mitochondrial gene expressions of *ppargc1a* and *cpt1a*, which are involved in fatty acid oxidation [28,29]. Notably, epigenetic regulation of CPT1a by fructose was reported [30]. At LF, the gene expression of *ppargc1a* or *cpt1a* did not show any heritability and was not correlated with the *cd36* gene expression. After the isocaloric low-carbohydrate, high-fat diet was given for one week, despite no changes in the gene expressions of *ppargc1a* or *cpt1a*, a significant positive correlation with the *cd36* gene expression was seen for both genes. Remarkably, at HF6, when the gene expressions of *ppargc1a* and *cpt1a* were significantly decreased, we observed a strong heritability for both genes (0.643 and 0.475, respectively) but the correlation with the *cd36* gene expression disappeared. Of note, the expression of both genes, namely, *ppargc1a* and *cpt1a*, were always significantly positively correlated with the most significant correlation at HF1. Taken together, this demonstrates that an isocaloric low-carbohydrate, high-fat diet acutely coordinated gene expression of fatty acid transport and metabolism of WAT; however, in the long term, adaptation was genetically determined.

A striking observation was the strong increase in the *slc2a5* gene expression at HF1 that persisted through to the end of the study at HF6. This increase appeared to be genetically determined since no heritability was observed at LF but was observed at HF1 (0.340) and HF6 (0.290). GLUT5 exclusively transports fructose across the cell membrane [31]. In humans, GLUT5 is most abundantly expressed in the jejunum and kidney but is also present in WAT, brain and skeletal muscle [31]. The gene expression of *slc2a5* is upregulated by an increase in its substrate fructose in jejunum [31] and WAT [32] and by increased insulin levels in skeletal muscle cells [33]. In our study, we could rule out the idea that increased fructose concentration in the systemic circulation was responsible for the increased *slc2a5* gene expression at HF1 and HF6 since the dietary intake of total carbohydrates, and specifically of fructose, was diminished at HF1 and HF6 compared with LF (Table S2). Furthermore, insulin levels that might affect the *slc2a5* gene expression did not change during the study (Table 2). Although the dietary intake of fructose was reduced once the low-carbohydrate, high-fat diet was started, it is likely that fructose entered the adipocyte since its carrier was upregulated. Targeted tracer studies using ^13^C-fructose in human Simpson–Golabi–Behmel syndrome adipocytes showed that fructose is metabolized to acetyl-CoA, which subsequently increases de novo fatty acid synthesis via FASN [34]. De novo lipogenesis mainly occurs in the liver but is also present in adipocytes [35] and is increased by diets high in carbohydrates and suppressed by high-fat diets [36]. The unexpected increase in the *fasn* gene expression seen at HF1 in our study seemed to be at least partially secondary due to increased acetyl-CoA resulting from increased carbohydrate flux into the adipocyte. Moreover, the *slc2a5* and *fasn* gene expressions were mildly positively correlated at LF and strongly positively correlated at HF1 and HF6.

The gene expression of *slc2a4* was also increased at HF1 and contributed to de novo lipogenesis (DNL) from glucose uptake. However, since glucose transport mediated by GLUT4 is mainly due to its increased translocation from intracellular vesicles to the plasma membrane after insulin stimulation [11], it is speculative whether changes in gene expression will reflect changes in transport activity. Of note, the *slc2a4* and *fasn* gene expressions were always very strongly correlated, with only little changes throughout the study.

GLUT1, which is a ubiquitously distributed glucose transporter [10,12], was significantly decreased at HF6 and showed a very strong heritability throughout the study, as we showed previously [37]. We were able to correlate the *slc2a1* gene expression with altered cognitive functions (for details, see [37]). Since the *slc2a1* gene expression was downregulated, never positively correlated with other genes and its heritability remained unchanged throughout the study, it seems unlikely that GLUT1 was a key molecule for alterations in fatty acid metabolism in adipose tissue after the consumption of an isocaloric low-carbohydrate, high-fat diet.

We observed an increase in the gene expression of *slc2a8*, which has a transport capacity for glucose and fructose [38], at HF1 and HF6. The function of GLUT8 is still not fully understood and it is not clear whether it is a plasma membrane transporter or is localized in intracellular compartments [39,40]. It was shown recently in a female mouse model that GLUT8 knockdown in liver reduces fructose-induced steatohepatosis and liver inflammation [41]. Another study in female mice demonstrated that GLUT8 mediates fructose-induced DNL in liver [42]. In our study, we observed no difference in the gene expressions of *il6* and *tnfa* in s.c. WAT at HF1 and HF6compared to LF which might be attributed to the absence of weight gain. While only a mild positive correlation of the *slc2a8* gene expression with the gene expression of *tnfa* and not of *il6* was observed at LF, a robust increase in correlation with both genes was seen at HF1, which persisted for the *tnfa* gene expression at HF6 only. The *slc2a8* gene expression was not correlated with the *fasn* gene expression at LF but was significantly positively correlated at LF1 and HF6. It was always strongly positively correlated with the *slc2a5* gene expression. Taken together, this indicates a regulatory effect of the isocaloric high-fat diet on fructose-dependent DNL in WAT. Further studies are needed to underpin these mechanisms.

For the gene expression of *adipoq*, we observed only a slight significant decrease at HF6, and for the gene expression of *pparg*, no differences were seen. This might reflect the stable body weight and adiposity due to the isocaloric study protocol. As expected, expressions of these genes were always strongly correlated throughout the study.

The metabolism of fatty acids and carbohydrates interacts with multiple organs, such as liver, skeletal muscle and adipose tissue. A limitation of this study was the missing data for hepatic and muscle metabolism after the switch to the isocaloric low-carbohydrate, high-fat diet. Another limitation was the termination of the study after a total of twelve weeks (six weeks for each diet). However, since the study was designed with a clear focus on adipocyte metabolism, the complexity of the design and the tight schedule of the participants was a limitation for additional experiments and a longer duration of the study.

The numbers of female and male participants were exactly balanced for dizygotic pairs of twins, whereas for monozygotic pairs of twins, more female than male participants were enrolled in this study. In order to evaluate gender- and age-related effects of the isocaloric low-carbohydrate, high-fat diet, studies with larger cohorts of exactly balanced gender and age of participants are needed.

In summary, we showed that an isocaloric low-carbohydrate, high-fat diet had acute and long-term regulatory and coordinating effects on gene expressions involved in the transport of fatty acids and carbohydrates and their downstream metabolism in human s.c. WAT. The gene expression involved in transmembrane fatty acid (*cd36* and *lpl*) and fructose transport (*slc2a5*) did not exhibit heritability when an isocaloric low-fat, high-carbohydrate diet was given. However, the temporary increase in *lpl* and the persistent increase in the *slc2a5* gene expression observed after the challenge of the isocaloric low-carbohydrate, high-fat diet were inherited, whereas the persistent decrease in the *cd36* gene expression was not. In contrast, the gene expression of intracellular metabolism was either always (*fasn* and *ppargc1a*) or never (*adipoq* and *pparg*) inherited.

## Figures and Tables

**Table 1:** Baseline characteristics of the participants.

	Monozygotic Twins	Dizygotic Twins
	(*n* = 68)	(*n* = 24)
Gender (F/M)	44/24	12/12
Age (years)	33.9 ± 14.9	24.5 ± 7.6
Body height (m)	1.71 ± 8.7	1.74 ± 11.6
Body weight (kg)	67.4 ± 11.9	68.0 ± 11.7
BMI (kg/m^2^)	23.0 ± 2.8	22.3 ± 2.2
Waist/hip ratio	0.82 ± 0.1	0.80 ± 0.1
Serum total cholesterol (mmol/L)	4.74 ± 0.9	4.15 ± 0.9
Serum LDL cholesterol (mmol/L)	2.84 ± 0.8	2.44 ± 0.6
Serum HDL cholesterol (mmol/L)	1.43 ± 0.3	1.24 ± 0.4
Serum triacylglycerol (mmol/L)	0.98 ± 0.4	1.01 ± 0.6
Serum-free fatty acids (mmol/L)	0.52 ± 0.2	0.52 ± 0.4
Fasted serum glucose (mg/dL)	86.2 ± 9.5	86.2 ± 6.4
Fasted serum insulin (mU/L)	5.30 ± 4.1	4.96 ± 2.2
HOMA IR index	1.16 ± 01.0	1.05 ± 0.5
HbA1c (%)	5.02 ± 0.4	0.52 ± 0.4

Values are given as mean ± SD.

**Table 2:** Body weight, BMI, serum lipids, blood glucose, insulin and HOMA-IR index after six weeks of the high-carbohydrate, low-fat diet (LF) and after one week (HF1) and six weeks (HF6) of the low-carbohydrate, high-fat diet.

	Monozygotic Twins		Dizygotic Twins			
	LF	HF1	HF6	LF	HF1	HF6
Body weight (kg)	66.4 ± 11.9	66.3 ± 11.9	66.8 ± 12.1	67.2 ± 11.3	67.1 ± 11.0	67.6 ± 11.5
BMI (kg/m^2^)	22.7 ± 2.9	22.6 ± 2.9	22.8 ± 2.9	22.1 ± 2.0	22.0 ± 1.9	22.2 ± 2.0
Total cholesterol (mmol/L)	4.42 ± 0.8	4.57 ± 0.8	4.83 ± 0.9 *	3.94 ± 0.8	4.18 ± 0.9	4.33 ± 0.8
LDL (mmol/L)	2.67 ± 0.7	2.76 ± 0.7	2.93 ± 0.8	2.38 ± 0.7	2.58 ± 0.7	2.65 ± 0.7
HDL (mmol/L)	1.30 ± 0.3	1.36 ± 0.4	1.46 ± 0.4	1.14 ± 0.3	1.21 ± 0.3	1.27 ± 0.3
Triglycerides (mmol/L)	0.97 ± 0.4	0.91 ± 0.3	0.90 ± 0.4	0.91 ± 0.5	0.86 ± 0.4	0.92 ± 0.4
Free fatty acids (mmol/L)	0.62 ± 0.2	0.55 ± 0.2	0.51 ± 0.2 §	0.56 ± 0.2	0.63 ± 0.2	0.47 ± 0.2 #
Glucose (mg/dL)	94.0 ± 13.2	93.6 ± 10.8	95.1 ± 10.7	93.9 ± 18.1	90.9 ± 12.4	90.9 ± 11.2
Insulin (mU/L)	4.78 ± 3.3	5.65 ± 4.0	5.30 ± 3.9	4.52 ± 2.7	5.29 ± 2.7	4.55 ± 2.8
HOMA-IR	1.06 ± 0.8	1.30 ± 1.0 $	1.26 ± 0.3 §	1.02 ± 0.5	1.17 ± 0.5 ¶	1.15 ± 0.3

Values are given as mean ± SD. Between-group differences were determined using one-way or repeated-measures ANOVA or the Kruskal–Wallis test. ¶ *p* < 0.05 and $ *p* < 0.01 for differences between LF and HF1; * *p* < 0.05 and § *p* < 0.01 for differences between LF and HF6; # *p* <0.01 for differences between HF1 and HF6 (*n* = 68 for monozygotic twins, *n* = 24 for dizygotic twins).

**Table 3:** Gene names, gene functions and their dietary regulations.

Gene Name and Function	Dietary Regulation		
	LF vs. HF1	LF vs. HF6	HF1 vs. HF6
*cd36*	↓↓↓	↓↓↓	↔
(hydrolysis of free fatty acids)			
*lpl*	↑	↔	↓
(lipid transport)			
*pdk4*	↓↓↓	↓↓↓	↓↓
(glycerol-3-phosphate generation)			
*fasn*	↑	↔	↓
(de novo fatty acid synthesis)			
*slc2a1*	↔	↓	↓
(glucose transport)			
*slc2a4*	↑	↔	↔
(glucose transport)			
*slc2a5*	↑↑↑	↑↑↑	↔
(fructose transport)			
*slc2a8*	↑↑	↑	↔
(glucose and fructose transport)			
*ppargc1a*	↔	↓	↓
(fatty acid oxidation)			
*cpt1a*	↔	↓↓	↓↓
(fatty acid oxidation)			
*adipoq*	↔	↓	↔
(adipogenesis)			
*pparg*	↔	↔	↔
(adipogenesis)			
*il6*	↔	↔	↔
(inflammation)			
*tnfa*	↔	↔	↔
(inflammation)			

Change in gene expression: ↑↑↑: strong increase, ↑↑: moderate increase, ↑: small increase, ↓↓↓: strong decrease, ↓↓: moderate decrease, ↓: small decrease, ↔: no change.

## Data Availability

Data available on request due to privacy restrictions.

## Supplementary Materials

The following supporting information can be downloaded from https://www.mdpi.com/article/10.3390/nu15102338/s1. Table S1: Human primer sequences used for quantitative real-time PCR analysis. Table S2: Intake of total carbohydrates, fructose, glucose and saccharose in grams (g) of three exemplary pairs of twins after six weeks on the isocaloric high-carbohydrate, low-fat diet (LF) and one week (HF1) and six (HF6) weeks on the isocaloric low-carbohydrate, high-carbohydrate diet.

## Abbreviations

*adipoq*: adiponectin, CD36: fatty acid translocase (FAT)/cluster of differentiation 36, *cpt1a*: carnitine palmitoyltransferase 1A, DNL: de novo lipogenesis, FFA: free fatty acid, FASN: fatty acid synthase, GLUT: glucose transporter, HOMA-IR: homeostasis model assessment of insulin resistance, *il6*: interleukin-6, LPL: lipoprotein lipase, PAL: physical activity level, *pdk4*: pyruvate dehydrogenase kinase, isozyme 4, *pparg*: peroxisome proliferator-activated receptor gamma, *ppargc1a*: peroxisome proliferator-activated receptor gamma, coactivator 1 alpha, REE: resting energy expenditure, *rpl32*: ribosomal protein L32, s.c.: subcutaneous, *slc2a1*: GLUT1, *slc2a4*: GLUT4, *slc2a5*: GLUT5, *slc2a8*: GLUT8, *tnfa*: tumor necrosis factor alpha, WAT: white adipose tissue.
