# Supplementary material for: An Isocaloric High-Fat Diet Regulates Partially Genetically Determined Fatty Acid and Carbohydrate Uptake and Metabolism in Subcutaneous Adipose Tissue of Lean Adult Twins
Source: Nutrients. 2023 May 16;15(10):2338. doi: 10.3390/nu15102338 (PMC10222154; doi:10.3390/nu15102338)
Supplement: Supplementary file 1 [file nutrients-15-02338-s001.zip › Supplement.pdf]

Table S1: Human primer sequences used for quantitative real-time PCR analysis.

Gene	Forward Primer	Reverse Primer
adipoq	GCAGTCTGTGGTTCTGATTCCA	GCAGTAGAACAGCTCCCAGCAA
cd36	GGAAGTGATGATGAACAGCAGC	ACTGTGTTGTCCTCAGCGTCCT
cpt1a	ATTATGCCATGGATCTGCTG	AGCGGAGCAGAGTGGAATC
fasn	AGACACTCGTGGGCTACAGCAT	ATGGCCTGGTAGGCGTTCT
il6	AGCCCTGAGAAAGGAGACATGTA	TCTGCCAGTGCCTCTTTGCT
lpl	ATGGCTGGACGGTAACAGGAA	TGACAGCCAGTCCACCACAAT
pdk4	CCCTGAGAATTATTGACCGCCT	AAGCCGTAACCAAAACCAGCC
pprarg	CAATTGAACAGCGCCGTGT	GTGCACTCCTCAATTTTCACCAA
ppargc1a	CAATGG AAGAGCGCCGTGT	GTGCACTCCTCAATTTCACCAA
rpl32	CAACGTCAAGGAGCTGGAAGT	TTGTGAGCGATCTCGGCAC
slc2a1	GGCCTTTTCGTTAACCGCTT	AGCATCTCAAAGGACTTGCCC
slc2a4	GCCGGACGTTTGACCAGA T	GGTGTTTCACCTCCTGCTCTA
slc2a5	GCATGGAGCAACAGGATCAGA	AGCAGCCACGTTGTACCCATAC
slc2a8	TTGAAGAGGCCAAGTTCAAGGA	TGACCACACCTGACAAGACCAG
tnf	GCCCATGTTGTAGCAAACCCT	ATGAGGTACAGGCCCTCTGATG

Gene abbreviations. *adipoq*: adiponectin, *cd36*: fatty acid translocase (FAT)/ cluster of differentiation 36, *cpt1a*: carnitine palmitoyltransferase 1A, *fasn*: fatty acid synthase, *il6*: interleukin-6, *lpl*: lipoprotein lipase, *pdk4*: pyruvate dehydrogenase kinase, isozyme 4, *pparg*: peroxisome proliferator-activated receptor gamma, *ppargc1a*: peroxisome proliferator-activated receptor gamma, coactivator 1 alpha, *rpl32*: ribosomal protein L32, *slc2a1*: GLUT1, *slc2a4*: GLUT4, *slc2a5*: GLUT5, *slc2a8*: GLUT8, *tnfa*: tumor necrosis factor alpha.

Table S2: Intake of total carbohydrates, fructose, glucose and saccharose in grams (g) of three exemplary pairs of twins after six weeks on the isocaloric high carbohydrate, low fat diet (LF) and one week (HF1) and six (HF6) weeks, respectively, on the isocaloric low carbohydrate, high carbohydrate diet.

	Intervention day	total carbohydrates (g)	fructose (g)	glucose (g)	saccharose (g)
Twin 1A	LF	391	40.3	24.5	91.8
	HF1	266	15.6	9.1	55.8
	HF6	268	16.1	10.0	55.1
Twin 1B	LF	362	39.0	24.1	78.7
	HF1	275	16.0	9.6	57.8
	HF6	264	15.7	10.1	56.8
Twin 2A	LF	279	24.8	19.0	41.0
	HF1	224	15.2	9.4	30.3
	HF6	209	14.9	8.3	23.6
Twin 2B	LF	292	23.3	18.6	46.0
	HF1	223	15.4	9.6	31.5
	HF6	201	16.5	8.5	20.2
Twin 3A	LF	298	33.4	21.6	64.1
	HF1	209	15.2	8.5	47.6
	HF6	222	13.5	8.4	49.9
Twin 3B	LF	313	37.0	24.0	60.9
	HF1	218	22.5	11.1	38.3
	HF6	219	22.3	11.2	38.7
